# Population-Based Prostate Cancer Screening With Magnetic Resonance Imaging or Ultrasonography: The IP1-PROSTAGRAM Study

**DOI:** 10.1001/jamaoncol.2020.7456

**Published:** 2021-02-11

**Authors:** David Eldred-Evans, Paula Burak, Martin J. Connor, Emily Day, Martin Evans, Francesca Fiorentino, Martin Gammon, Feargus Hosking-Jervis, Natalia Klimowska-Nassar, William McGuire, Anwar R. Padhani, A. Toby Prevost, Derek Price, Heminder Sokhi, Henry Tam, Mathias Winkler, Hashim U. Ahmed

**Affiliations:** 1 Imperial Prostate, Division of Surgery, Department of Surgery and Cancer, Faculty of Medicine, Imperial College London, London, United Kingdom; 2 Department of Urology, Imperial College Healthcare National Health Service (NHS) Trust, London, United Kingdom; 3 Imperial Clinical Trials Unit, Imperial College London, London, United Kingdom; 4 Division of Surgery, Department of Surgery and Cancer, Faculty of Medicine, Imperial College London, London, United Kingdom; 5 Paul Strickland Scanner Centre, Mount Vernon Hospital, Middlesex, United Kingdom; 6 Department of Radiology, The Hillingdon Hospitals NHS Foundation Trust, London, United Kingdom; 7 Department of Radiology, Imperial College Healthcare NHS Trust, London, United Kingdom

## Abstract

**Importance:**

Screening for prostate cancer using prostate-specific antigen (PSA) testing can lead to problems of underdiagnosis and overdiagnosis. Short, noncontrast magnetic resonance imaging (MRI) or transrectal ultrasonography might overcome these limitations.

**Objective:**

To compare the performance of PSA testing, MRI, and ultrasonography as screening tests for prostate cancer.

**Design, Setting, and Participants:**

This prospective, population-based, blinded cohort study was conducted at 7 primary care practices and 2 imaging centers in the United Kingdom. Men 50 to 69 years of age were invited for prostate cancer screening from October 10, 2018, to May 15, 2019.

**Interventions:**

All participants underwent screening with a PSA test, MRI (T2 weighted and diffusion), and ultrasonography (B-mode and shear wave elastography). The tests were independently interpreted without knowledge of other results. Both imaging tests were reported on a validated 5-point scale of suspicion. If any test result was positive, a systematic 12-core biopsy was performed. Additional image fusion–targeted biopsies were performed if the MRI or ultrasonography results were positive.

**Main Outcomes and Measures:**

The main outcome was the proportion of men with positive MRI or ultrasonography (defined as a score of 3-5 or 4-5) or PSA test (defined as PSA ≥3 μg/L) results. Key secondary outcomes were the number of clinically significant and clinically insignificant cancers detected if each test was used exclusively. Clinically significant cancer was defined as any Gleason score of 3+4 or higher.

**Results:**

A total of 2034 men were invited to participate; of 411 who attended screening, 408 consented to receive all screening tests. The proportion with positive MRI results (score, 3-5) was higher than the proportion with positive PSA test results (72 [17.7%; 95% CI, 14.3%-21.8%] vs 40 [9.9%; 95% CI, 7.3%-13.2%]; *P* < .001). The proportion with positive ultrasonography results (score, 3-5) was also higher than the proportion of those with positive PSA test results (96 [23.7%; 95% CI, 19.8%-28.1%]; *P* < .001). For an imaging threshold of score 4 to 5, the proportion with positive MRI results was similar to the proportion with positive PSA test results (43 [10.6%; 95% CI, 7.9%-14.0%]; *P* = .71), as was the proportion with positive ultrasonography results (52 [12.8%; 95% CI, 9.9%-16.5%]; *P* = .15). The PSA test (≥3 ng/mL) detected 7 clinically significant cancers, an MRI score of 3 to 5 detected 14 cancers, an MRI score of 4 to 5 detected 11 cancers, an ultrasonography score of 3 to 5 detected 9 cancer, and an ultrasonography score of 4 to 5 detected 4 cancers. Clinically insignificant cancers were diagnosed by PSA testing in 6 cases, by an MRI score of 3 to 5 in 7 cases, an MRI score of 4 to 5 in 5 cases, an ultrasonography score of 3 to 5 in 13 cases, and an ultrasonography score of 4 to 5 in 7 cases.

**Conclusions and Relevance:**

In this cohort study, when screening the general population for prostate cancer, MRI using a score of 4 or 5 to define a positive test result compared with PSA alone at 3 ng/mL or higher was associated with more men diagnosed with clinically significant cancer, without an increase in the number of men advised to undergo biopsy or overdiagnosed with clinically insignificant cancer. There was no evidence that ultrasonography would have better performance compared with PSA testing alone.

## Key Points

**Question:**

In men invited to undergo screening for prostate cancer with magnetic resonance imaging (MRI), ultrasonography, and prostate-specific antigen testing, what is the prevalence of positive test results, rates of biopsy, and detection of prostate cancer?

**Findings:**

In this cohort study in which 408 men underwent 3 screening tests, an MRI score of 4 or 5 was associated with improved detection of clinically significant prostate cancer without an increase in the number of men who underwent biopsy or were overdiagnosed with clinically insignificant prostate cancer if prostate-specific antigen testing alone was used. Ultrasonography was not associated with improved screening performance.

**Meaning:**

These findings suggest that a short, noncontrast MRI may have favorable performance characteristics as a community-based screening test.

## Teaser

This cohort study of men undergoing prostate cancer testing compares the performance of prostate-specific antigen testing, magnetic resonance imaging, and ultrasonography as screening tests for prostate cancer.

## Introduction

A population screening program for prostate cancer is not currently recommended.^1,2^ Men can request a serum prostate-specific antigen (PSA) test in primary care provided they are informed of the benefits and risks. A PSA test can reduce the relative risk of mortality from prostate cancer by approximately 20% when repeated at 2- to 4-year intervals.^3^ However, it will miss a proportion of clinically significant prostate cancers (underdiagnosis) while identifying clinically insignificant prostate cancers (overdiagnosis) that do not require treatment.^4,5^

Imaging has been the technique of choice in lung and breast cancer population screening programs. In prostate cancer, various imaging modalities are available, but evidence is limited to use in men within secondary care rather than the general population.^6,7^ Multiparametric, contrast-enhanced magnetic resonance imaging (MRI) is a diagnostic test with good accuracy for detection of clinically significant cancer in men referred to the hospital with an elevated PSA level.^8,9,10^ Recently, biparametric, noncontrast (short) MRI protocols have been developed that offer shorter scanning times with a favorable diagnostic performance.^11^

An alternative, inexpensive, and more widely available test is ultrasonography. Recently, the combination of standard B-mode imaging with other ultrasonographic modalities, such as shear wave elastography (SWE), has shown promise.^12^ Shear wave elastography provides a quantitative measurement of suspicious areas within the prostate based on tissue elasticity.

The Imperial Prostate 1 Prostate Cancer Screening Trial Using Imaging (IP1-PROSTAGRAM) study was established to determine the performance of these imaging tests for prostate cancer screening. We report the primary outcomes comparing the proportion of men advised to undergo biopsy when screened with short MRI, ultrasonography, or PSA test. We also report the subsequent number of men diagnosed with clinically significant and clinically insignificant prostate cancer by each test.

## Methods

### Study Design

The IP1-PROSTAGRAM study was a prospective, blinded, population-based screening study for prostate cancer approved by the UK National Research Ethics Committee and conducted in accordance with Good Clinical Practice guidelines and the Declaration of Helsinki.^13^ All participants provided written informed consent. All data were pseudo-anonymized. The study was managed by the Imperial Clinical Trials Unit overseen by an independent trial steering committee. The study followed the Strengthening the Reporting of Observational Studies in Epidemiology (STROBE) reporting guideline.^14^

### Participants

Between October 10, 2018, and May 15, 2019, men were invited to participate in the study through 7 primary care practices that were part of the UK National Institute for Health Research Clinical Research Network. To address the low response rate of racial/ethnic minorities in previous population-based screening trials, a dedicated direct-to-community recruitment strategy was developed. This strategy was implemented at study sites with high racial/ethnic diversity and involved a range of community outreach activities, such as posters displayed in community locations and support from local community leaders (eMethods in the Supplement). Race/ethnicity was classified based on fixed categories and was self-identified. Men 50 to 69 years of age with a life expectancy of at least 10 years were invited to participate provided they had not undergone PSA testing or prostate MRI in the previous 2 years; had not had a urinary infection or prostatitis in the previous 6 months; and did not have a history of prostate biopsy, prostate cancer, or any contraindication to MRI (eMethods in the Supplement).

### Procedures

#### Screening Tests

All participants who met the eligibility criteria underwent serum PSA testing, short MRI, and ultrasonography. The MRI and ultrasonography results were independently assessed by different reporters blinded to the PSA level and the demographic and clinical information apart from age. To minimize attrition bias, participants were blinded to the details of screening test results. Participants with positive results were informed that 1 or more test results were positive but not informed which test result was positive until study completion. All men were unblinded on study completion, including those men who tested negative on all screening tests.

The short MRI protocol had an acquisition time of approximately 15 minutes. It included T2-weighted and diffusion-weighted imaging with multiple b-values (0, 150, 400, 1000, and 1500 s/mm^2^) (eMethods in the Supplement). The protocol did not include gadolinium contrast enhancement^15,16^ and was performed using one 1.5- and one 3.0-T scanner (Aera 1.5T or Siemens Magnetom Verio 3T) with pelvic phased-array coils at 2 sites. Each site’s MRI scans were centrally reviewed for quality before recruitment. The MRI scans were interpreted by 2 radiologists (H.S. and H.T.), 1 at each site, experienced in reporting prostate MRI scans (eMethods in the Supplement).

Ultrasonography included B-mode and SWE acquired on an ultrafast scanner (Aixplorer, SuperSonic Imagine) using an end-fire endorectal probe (SuperEndocavity SE12-3, Supersonic Imagine). The examinations were performed at 2 sites by 2 urologists experienced in transrectal ultrasonography (eMethods in the Supplement). The quality of ultrasonographic images from each site was assessed before commencing the study. The ultrasonography assessment followed a predefined scanning protocol in which the prostate and seminal vesicles were captured systematically in the axial and sagittal planes. All ultrasonographic images were reported by the operator who performed the scan at each site.

Both MRI scans and ultrasonographic images were independently reported using a scale of 1 to 5, with higher numbers indicating greater likelihood of clinically significant prostate cancer. The MRI scans were scored using the Prostate Imaging–Reporting and Data System, version 2^17^ revised for scoring without the contrast-enhanced sequence (eFigure 1 in the Supplement). The B-mode ultrasonography was scored using a validated 5-point scoring system,^18^ and elastography was scored using the World Federation for Ultrasound in Medicine and Biology guidelines adapted for SWE^19^ (eMethods in the Supplement). Both MRI and ultrasonography scores were dichotomized at a score of 3 (equivocal) and 4 (likely clinically significant cancer) to create 2 thresholds, a score equal to or above which defined a positive MRI or ultrasonography result (eFigure 2 in the Supplement). To assess interobserver agreement, 20% of the MRI scans and ultrasonographic images, stratified by score to ensure a representative sample, were selected at random from both sites and reviewed by a second reporter (A.R.P. or M.J.C.).

During the screening visit, participants were reviewed by a urologist (D.E.E.) to exclude those with a urinary tract infection and perform a digital rectal examination. The digital rectal examination was only performed after the blood sample for PSA testing had been collected. Serum PSA testing was performed at a single laboratory (Imperial College National Health Service Health Care Trust) and measured using an automated chemiluminescent microparticle immunoassay analyzer (Abbott Diagnostics) referenced against the World Health Organization First International Standard for PSA (90:10), coded 96/670.^20^ The PSA level was dichotomized as a positive (≥3 ng/mL) or negative (<3 ng/mL) result (to convert values to micrograms per liter, multiply by 1).

#### Biopsy

A biopsy was recommended in the presence of any positive test result. Men underwent a transperineal, systematic, 12-core biopsy. Additional image fusion–targeted biopsies of all MRI and ultrasonography lesions were also performed with each target placed in a separate biopsy pot to be reported individually. If both MRI and ultrasonography lesions were present, the order of targeted biopsy was allocated by computer-generated random number sequence and block randomization.

All biopsies were performed using a biplanar transrectal ultrasonography machine (Hitachi Prerius, Hitachi Medical Corporation) mounted to a brachytherapy stepper and grid (Civco). Image fusion biopsies were performed with software-assisted registration (BiopSee, MedCom GmbH). Reporting radiologists were not present during the biopsy to ensure the reference test remained independent of the index test. All biopsy specimens were centrally reviewed by expert uropathologists according to the International Society of Urological Pathology guidelines.^21^

### Outcome Measures

We determined the proportions of men with positive MRI and ultrasonography results at different radiologic scores to define a positive test result (score, 3-5 or 4-5) and compared them with men with positive PSA test results (≥3 ng/mL). Secondary outcomes were the number of men with clinically significant and clinically insignificant cancers by each screening test. The primary histologic definition used a target condition on systematic or targeted biopsy of any amount of a Gleason score of 3+4 or higher. Adverse events were recorded until the last study visit.

### Statistical Analysis

The sample size estimate for the primary outcome was calculated using the formula described by Naing et al^22^ with the estimate of the prevalence of positive MRI results determined from the literature (eMethods in the Supplement). A target of 406 participants was calculated to provide a ±5% precision estimate at a 2-sided significance level of *P* < .05, allowing for a 10% dropout rate.

All analyses were completed according to a prespecified statistical analysis plan, which was agreed on before database lock and commencement of any analysis. All analyses were undertaken at the patient level. The primary analysis included all eligible participants who completed at least 1 screening test. For each MRI and ultrasonography threshold, the proportion of men with positive test results was estimated with the corresponding 95% CI. McNemar tests were used for the head-to-head comparisons of the paired proportions of positive screening results between MRI and PSA and between ultrasonography and PSA. For participants who underwent biopsy, the number of men diagnosed with clinically significant and clinically insignificant cancers was reported.

Interobserver agreement was evaluated using percentage agreement and the Cohen κ statistic for a random sample of double reported MRI and ultrasonography results. The random sample was obtained by dividing all MRI scans and ultrasonographic images into 3 groups according to the primary readers’ Prostate Imaging–Reporting and Data System or ultrasonography score: group A included those with negative results (score of 1 or 2), group B included those with indeterminate results (score 3), and group C were those with positive results (score of 4 or 5). Twenty-six images were randomly selected from each group so that a total of 78 (equivalent to 20% of each) were double reported. All analyses were performed with Stata software, version 15.1 (StataCorp LLC).

## Results

**Figure 1.**
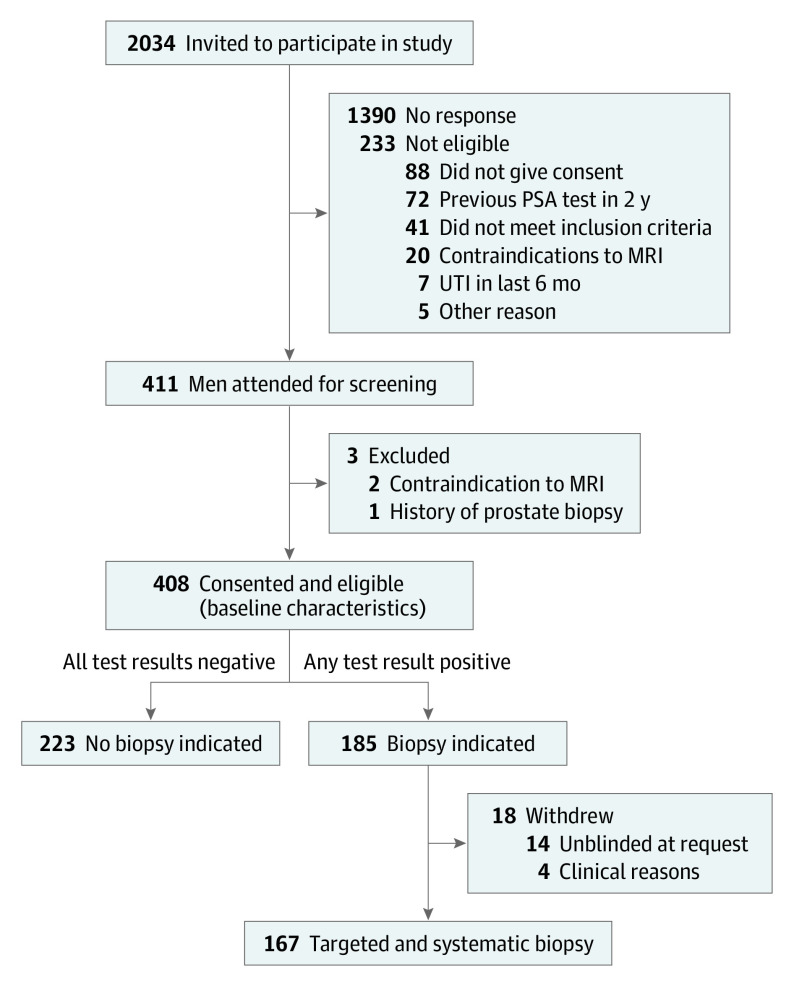
Screening, Recruitment, and Flow of Participants MRI indicates magnetic resonance imaging; PSA, prostate-specific antigen; UTI, urinary tract infection.

A total of 2034 men were invited to participate in the study; 1707 (83.9%) received invitations from their general physician and 327 (16.1%) through the direct-to-community recruitment strategy. The recruitment target was met 19 months ahead of schedule. A total of 411 participants attended screening, and 408 consented and were eligible to receive all screening tests (Figure 1). Baseline characteristics for all eligible participants are presented in Table 1. A total of 403 completed all screening tests, of whom 310 received the tests on the same day (interval between tests: median, 0 days; range, 0-76 days). The MRI was performed at 3 T in 338 patients (82.8%) and 1.5 T in 70 patients (17.2%). Three participants did not tolerate the ultrasonography, and 2 participants did not complete the MRI.

**Table 1.** Baseline Characteristics of the Study Participants (N = 408)

Characteristic	No. (%) of participants
Age group, y	
50-54	140 (34.3)
55-59	127 (31.1)
60-64	85 (20.8)
65-69	56 (13.7)
Racial/ethnic group	
White	155 (38.0)
Black	132 (32.4)
Asian	94 (23.0)
Other	18 (4.4)
Mixed race	9 (2.2)
Index of multiple deprivation quintile	
1 (Least deprived)	54 (13.2)
2	109 (26.7)
3	137 (33.6)
4	62 (15.2)
5 (Most deprived)	44 (10.8)
Unknown	2 (0.5)
Charlson comorbidity index	
0 (None)	324 (79.4)
1 (Mild)	49 (12.0)
≥2 (Severe)	13 (3.2)
Score unknown	22 (5.4)
First-degree relative with prostate cancer	
Yes	43 (10.5)
No	360 (88.2)
Unknown	5 (1.2)
5α-Reductase inhibitors	
Yes	1 (0.2)
No	407 (99.8)
IPSS score	
Mild (≤7)	277 (67.9)
Moderate	112 (27.5)
Severe	10 (2.5)
Unknown	9 (2.2)
Previous PSA test^a^	
No	291 (71.3)
Yes	
2-3 y Ago	30 (7.4)
4-5 y Ago	41 (10.0)
>5 y Ago	26 (6.4)
Date not known	6 (1.5)
Unknown	14 (3.4)
Digital rectal examination findings	
Normal	387 (94.9)
Abnormal	18 (4.4)
Unknown	3 (0.7)

Abbreviations: IPSS, International Prostate Symptom Score; PSA, prostate-specific antigen.

^a^ Exclusion criteria included any PSA test in the past 2 years.

Of 406 patients, 72 (17.7%; 95% CI, 14.0%-21.8%) had an MRI score of 3 to 5, and 40 (9.9%; 95% CI, 7.3%-13.2%) had a positive PSA test result (*P* < .001). Of 405 patients, 96 (23.7%; 95% CI, 19.8%-28.1%) had a positive ultrasonography result (score of 3-5), which was also higher than the PSA result (*P* < .001). If the threshold for MRI and ultrasonography was set at a score of 4 to 5, 43 (10.6%; 95% CI, 7.9%-14.0%) of 406 patients had positive MRI results, which was similar to the PSA results (*P* = .71), and 52 (12.8%; 95% CI, 9.9%-16.5%) of 405 patients had positive ultrasonography results, which was also similar to the PSA results (*P* = .15).

In total, 37 prostate cancers were identified on combined targeted and systematic biopsy (17 clinically significant and 20 clinically insignificant). For clinically significant cancers, PSA testing (≥3 ng/mL) detected 7 cases, MRI score of 3 to 5 detected 14 cases, and MRI score of 4 to 5 detected 11 cases (Table 2). Ultrasonography score of 3 to 5 detected 9 cases, and ultrasonography score of 4 to 5 detected 4 cases. For clinically insignificant cancers, PSA testing (≥3 ng/mL) detected 6 cases, MRI score of 3 to 5 detected 7 cases, MRI score of 4 to 5 detected 5 cases, ultrasonography score of 3 to 5 detected 13 cases, and ultrasonography score of 4 to 5 detected 7 cases (eFigures 3-8 and eTable 1 in the Supplement).

**Table 2.** Number of Positive Test Results and Cancers Detected by MRI, Ultrasonography, and PSA testing

Variable	MRI		Ultrasonography		PSA (≥3 ng/mL)
	Score 3-5	Score 4-5	Score 3-5	Score 4-5	
Positive test result	72	43	96	52	40
Biopsy result					
Significant cancer^a^	14	11	9	4	7
Insignificant cancer^b^	7	5	13	7	6
Benign	44	22	63	35	23
Withdrew^c^	7	5	11	6	4

Abbreviations: MRI, magnetic resonance imaging; PSA, prostate-specific antigen.

SI conversion factor: to convert PSA to micrograms per liter, multiply by 1.

^a^ Significant cancer is defined as a Gleason score of 3+4 or higher (International Society of Urological Pathology score ≥2) (primary histologic definition of clinical significance).

^b^ Insignificant cancer is defined as a Gleason score of 3+3 (International Society of Urological Pathology score of 1).

^c^ The number of men who withdrew after being advised to have a biopsy.

**Figure 2.**
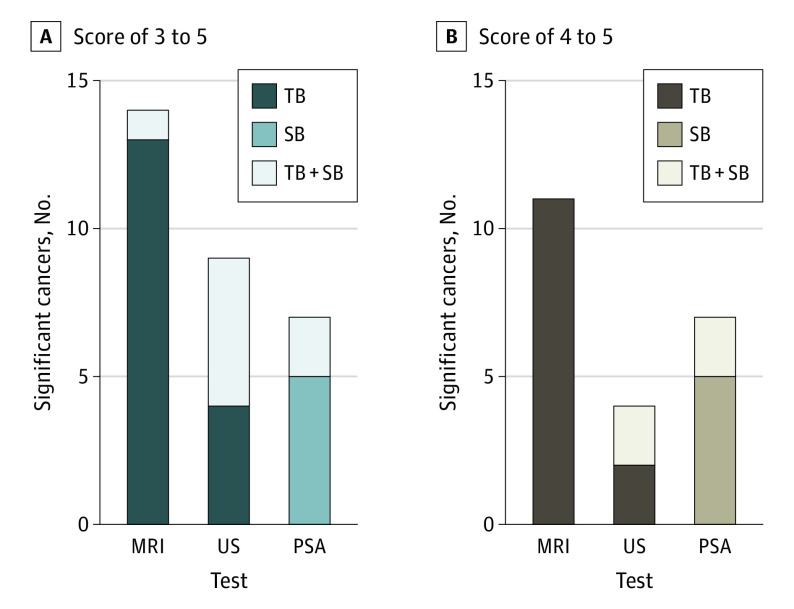
Comparison of Detected Clinically Significant Cancers by Each Screening Test According to Magnetic Resonance Imaging (MRI) and Ultrasonography (US) Threshold A, Clinically significant cancer is defined as a Gleason score of 3+4 or higher (International Society of Urological Pathology score ≥2). Positive test results are defined as a prostate-specific antigen (PSA) level of 3 ng/mL or higher, an MRI score of 3 to 5, and a US score of 3 to 5. The PSA levels for the 8 men with MRI scores of 3 to 5 and a PSA level less than 3 ng/mL (to convert to micrograms per liter, multiply by 1) are provided in eTable 3 in the Supplement. B, Clinically significant cancer is defined as a Gleason score of 3+4 or higher (International Society of Urological Pathology score ≥2). TB indicates targeted biopsy; SB, systematic biopsy.

The image fusion–targeted biopsy detected significant cancer for MRI score of 3 to 5 in 13 cases, MRI score of 4 to 5 in 11 cases, ultrasonography score of 3 to 5 in 4 cases, and ultrasonography score of 4 to 5 in 2 cases. The systematic biopsy detected additional significant cancer for MRI score of 3 to 5 in 1 case, ultrasonography score of 3 to 5 in 5 cases, and ultrasonography score of 4 to 5 in 2 cases. No cases of additional significant cancer were detected by systematic biopsy for MRI score of 4 to 5. In the PSA group, the systematic biopsy detected 5 cases of significant cancer, and 2 additional cases were found on targeted biopsy because of a concurrent suspicious area on imaging (Figure 2).

Seventy-eight MRI scans and ultrasonographic images underwent double-reporting by a reporter with no knowledge of the serum PSA level, original MRI or ultrasonography report, or biopsy outcome. The percentage agreements were 61.5% for an MRI score of 3 to 5, 70.5% for an MRI score of 4 to 5, 76.9% for an ultrasonography score of 3 to 5, and 65.4% for an ultrasonography score of 4 to 5. The κ values were 0.27 (fair) for an MRI score of 3 to 5, 0.26 (fair) for an MRI score of 4 to 5, 0.50 (moderate) for an ultrasonography score of 3 to 5, and 0.33 for an ultrasonography score of 4 to 5 (fair) (eTables 2 and 3 in the Supplement).

No serious adverse events occurred. Of the adverse events that occurred, 3 were associated with MRI, 5 with ultrasonography, and 1 with PSA. The most common adverse event was procedure-related pain or anxiety (4 events associated with ultrasonography and 2 events associated with MRI) (eTable 4 in the Supplement).

## Discussion

In this cohort study, with MRI and ultrasonography scores of 3 to 5, the proportion of men advised to undergo biopsy was higher than the proportion advised to undergo biopsy after a PSA test result of 3 or higher. Magnetic resonance imaging was associated with detection of approximately twice as many clinically significant cancers. With the use of the higher MRI and ultrasonography scores of 4 and 5 to denote a positive screening test result, similar numbers of men were advised to undergo biopsy and PSA testing, with MRI potentially detecting more clinically significant cancers and ultrasonography detecting fewer clinically significant cancers than PSA testing.

To our knowledge, IP1-PROSTAGRAM is the first study to test the role of new imaging techniques as screening tests for prostate cancer compared with PSA testing. The key strengths of this study were the use of a blinded, paired-cohort design to evaluate 2 different imaging tests with neither using intravenous contrast. A recommendation for biopsy was made if any one of the independent test results was positive, without men being made aware of which was positive. Furthermore, by setting imaging score thresholds leading to a recommendation for biopsy at 3 or greater, it was possible to formally evaluate which imaging score threshold might be used for future confirmatory studies of an imaging-based screening strategy for prostate cancer. Of importance, unlike other studies,^23,24^ the imaging screening tests were used irrespective of PSA level.

The paired, screen-positive design in IP1-PROSTAGRAM provided a valuable opportunity to reevaluate PSA, using a threshold of 3 ng/mL, with contemporary prostate biopsy techniques that incorporated image fusion–targeted biopsies alongside systematic sampling. Other trials that evaluated new biomarkers for screening have used a PSA cutoff below which the new biomarker was not applied.^23,24^ The early PSA studies^25,26^ provided historic evidence that a threshold of 3 ng/mL will miss clinically significant cancers, and in the UK Cluster Randomized Trial of PSA Testing for Prostate Cancer,^4^ 68 (46.6%) of 146 men dying of prostate cancer had a PSA level less than 3 ng/mL.

Population-based screening tests should have a well-defined and agreed-on cutoff point that is set to maximize detection of potentially life-threatening cancers while minimizing harms from false-positive results and overdiagnosis.^27^ The findings of this study indicate that an MRI score of 4 or 5 may provide a better balance between the potential benefits and harms of screening. These findings suggest that ultrasonography, at either score threshold, would not provide an improved trade-off in performance compared with PSA testing.

Even short MRI has considerable additional resources and cost compared with PSA testing and ultrasonography. There are examples of highly accurate, more expensive screening tests that are cost-effective when applied at longer intervals. In colorectal screening, computed tomographic colonoscopy and flexible sigmoidoscopy are more sensitive than the fecal occult blood test and are cost-effective when applied at intervals of 5 to 10 years.^28^ Additional studies of prostate imaging at different screening intervals are needed.

Because of the low prevalence of cancers in the general population, IP1-PROSTAGRAM was not powered to evaluate differences in detection rates of clinically significant cancer between each screening test. As a result, a formal statistical comparison of detection rates was not prespecified in the statistical analysis plan. A future study is needed to definitively prove these differences as a substantially larger paired-cohort study or in a randomized comparative design.

If the role of MRI is to be advanced for screening, further work will be required to address the high interobserver variability. Future clinical trials may benefit from consensus double interpretation of MRI, which improves the diagnostic accuracy of screening mammography.^29^ In addition, the low level of agreement seen in this study could be attributable to the lack of a specific MRI scoring system in this population. The Prostate Imaging–Reporting and Data System, version 2 scoring system was developed for evaluating multiparametric MRI in men referred with suspected prostate cancer to secondary care, so its application in the general male population with much lower cancer prevalence may require specific modifications.

### Limitations

This study has limitations. First, although the paired design is routine for screening studies, not all participants received a biopsy. This approach was necessary because of the ethical concerns of performing a prostate biopsy in healthy men from the general population when all test results were, by definition, negative. Second, the direct-to-community recruitment strategy to increase participation of racial/ethnic minorities might introduce selection bias. This strategy was chosen in response to previous prostate cancer screening studies using PSA that failed to adequately recruit racial/ethnic minorities. Third, both sites had experience in prostate MRI and ultrasonography, which limits the generalizability of the results. Fourth, the ultrasonography protocol did not include contrast enhancement, which may have reduced its performance characteristics. During the design phase of the study, it was decided that both tests should not include intravenous contrast agents because this was unlikely to be acceptable as a screening test.

## Conclusions

This study suggests that when screening the general population for prostate cancer, MRI using a score of 4 or 5 to define a positive test result, compared with PSA testing alone at a level of 3 ng/mL or higher, might lead to more men being diagnosed with clinically significant cancer, without increasing the number of men advised to undergo biopsy or overdiagnosed with clinically insignificant cancer. The findings also suggest that ultrasonography is unlikely to have better performance characteristics compared with PSA testing alone.

## Notes

Supplement. **eMethods.** Supplemental Methods **eTable 1.** PSA Levels <3ng/ml in Men With an MRI Score 3-5 & Significant Cancer on Biopsy **eTable 2.** Agreement in MRI Scores Between Primary Readers and a Secondary Reader **eTable 3.** Agreement in US Scores Between Primary Reader and a Secondary Reader **eTable 4.** Adverse Events **eFigure 1.** A Screen-Negative MRI **eFigure 2.** A Screen-Positive MRI and Screen-Negative Ultrasound **eFigure 3.** Venn Diagrams Demonstrating Overlap of Clinically Significant Cancers Identified by Each Screening Test **eFigure 4.** Flowchart of MRI Score 3-5 and Detection of Clinically Significant Cancer **eFigure 5.** Flowchart of MRI Score 4-5 and Detection of Clinically Significant Cancer **eFigure 6.** Flowchart of US Score 3-5 and Detection of Clinically Significant Cancer **eFigure 7.** Flowchart of US Score 4-5 and Detection of Clinically Significant Cancer **eFigure 8.** Flowchart of PSA ≥ 3ng and Detection of Clinically Significant Cancer

## References

[1] US Preventive Services Task Force. Final Recommendation Statement: Prostate Cancer: Screening. May 2018. Accessed February 29, 2020. https://www.uspreventiveservicestaskforce.org/uspstf/document/RecommendationStatementFinal/prostate-cancer-screening

[2] UK National Screening Committee (NSC). The UK NSC Recommendation on Prostate Cancer Screening/PSA Testing in Men Over the Age of 50. Accessed February 29, 2020. https://legacyscreening.phe.org.uk/prostatecancer

[3] Hugosson J, Roobol MJ, Månsson M, ; ERSPC investigators. A 16-yr follow-up of the European randomized study of screening for prostate cancer. Eur Urol. 2019;76(1):43-51. doi:10.1016/j.eururo.2019.02.009 30824296 PMC7513694

[4] Martin RM, Donovan JL, Turner EL, ; CAP Trial Group. Effect of a low-intensity PSA-based screening intervention on prostate cancer mortality: the CAP randomized clinical trial. JAMA. 2018;319(9):883-895. doi:10.1001/jama.2018.0154 29509864 PMC5885905

[5] Thompson IM, Ankerst DP, Chi C, . Operating characteristics of prostate-specific antigen in men with an initial PSA level of 3.0 ng/ml or lower. JAMA. 2005;294(1):66-70. doi:10.1001/jama.294.1.66 15998892

[6] Eldred-Evans D, Tam H, Sokhi H, Padhani AR, Winkler M, Ahmed HU. Rethinking prostate cancer screening: could MRI be an alternative screening test? Nat Rev Urol. 2020;17(9):526-539. doi:10.1038/s41585-020-0356-2 32694594

[7] Nam RK, Wallis CJ, Stojcic-Bendavid J, . A pilot study to evaluate the role of magnetic resonance imaging for prostate cancer screening in the general population. J Urol. 2016;196(2):361-366. doi:10.1016/j.juro.2016.01.114 26880413

[8] Ahmed HU, El-Shater Bosaily A, Brown LC, ; PROMIS study group. Diagnostic accuracy of multi-parametric MRI and TRUS biopsy in prostate cancer (PROMIS): a paired validating confirmatory study. Lancet. 2017;389(10071):815-822. doi:10.1016/S0140-6736(16)32401-1 28110982

[9] Drost FH, Osses DF, Nieboer D, . Prostate MRI, with or without MRI-targeted biopsy, and systematic biopsy for detecting prostate cancer. Cochrane Database Syst Rev. 2019;4:CD012663. doi:10.1002/14651858.CD012663.pub2 31022301 PMC6483565

[10] Kasivisvanathan V, Rannikko AS, Borghi M, ; PRECISION Study Group Collaborators. MRI-targeted or standard biopsy for prostate-cancer diagnosis. N Engl J Med. 2018;378(19):1767-1777. doi:10.1056/NEJMoa1801993 29552975 PMC9084630

[11] van der Leest M, Israël B, Cornel EB, . High diagnostic performance of short magnetic resonance imaging protocols for prostate cancer detection in biopsy-naïve men: the next step in magnetic resonance imaging accessibility. Eur Urol. 2019;76(5):574-581. doi:10.1016/j.eururo.2019.05.029 31167748

[12] Sang L, Wang X-M, Xu D-Y, Cai Y-F. Accuracy of shear wave elastography for the diagnosis of prostate cancer: a meta-analysis. Sci Rep. 2017;7(1):1949. doi:10.1038/s41598-017-02187-0 28512326 PMC5434001

[13] World Medical Association. World Medical Association Declaration of Helsinki: ethical principles for medical research involving human subjects. JAMA. 2013;310(20):2191-2194. doi:10.1001/jama.2013.281053 24141714

[14] von Elm E, Altman DG, Egger M, Pocock SJ, Gøtzsche PC, Vandenbroucke JP; STROBE Initiative. The Strengthening the Reporting of Observational Studies in Epidemiology (STROBE) statement: guidelines for reporting observational studies. Ann Intern Med. 2007;147(8):573-577. doi:10.7326/0003-4819-147-8-200710160-00010 17938396

[15] Brizmohun Appayya M, Adshead J, Ahmed HU, . National implementation of multi-parametric magnetic resonance imaging for prostate cancer detection - recommendations from a UK consensus meeting. BJU Int. 2018;122(1):13-25. doi:10.1111/bju.14361 29699001 PMC6334741

[16] Barentsz JO, Weinreb JC, Verma S, . Synopsis of the PI-RADS v2 guidelines for multiparametric prostate magnetic resonance imaging and recommendations for use. Eur Urol. 2016;69(1):41-49. doi:10.1016/j.eururo.2015.08.038 26361169 PMC6364687

[17] Weinreb JC, Barentsz JO, Choyke PL, . PI-RADS Prostate Imaging-Reporting and Data System: 2015, version 2. Eur Urol. 2016;69(1):16-40. doi:10.1016/j.eururo.2015.08.052 26427566 PMC6467207

[18] Xie SW, Wang YQ, Dong BJ, . A nomogram based on a TRUS five-grade scoring system for the prediction of prostate cancer and high grade prostate cancer at initial TRUS-guided biopsy. J Cancer. 2018;9(23):4382-4390. doi:10.7150/jca.27344 30519343 PMC6277649

[19] Barr RG, Cosgrove D, Brock M, . WFUMB guidelines and recommendations on the clinical use of ultrasound elastography, part 5: prostate. Ultrasound Med Biol. 2017;43(1):27-48. doi:10.1016/j.ultrasmedbio.2016.06.020 27567060

[20] National Institute for Biological Standards and Control. WHO International Standard Prostate Specific Antigen (90:10) (NIBSC code: 96/670). Accessed February 29, 2020. https://www.nibsc.org/documents/ifu/96-670.pdf2011

[21] Epstein JI, Egevad L, Amin MB, Delahunt B, Srigley JR, Humphrey PA; Grading Committee. The 2014 International Society of Urological Pathology (ISUP) Consensus Conference on Gleason Grading of Prostatic Carcinoma: definition of grading patterns and proposal for a new grading system. Am J Surg Pathol. 2016;40(2):244-252. 26492179 10.1097/PAS.0000000000000530

[22] Naing L, Winn T, Rusli B. Practical issues in calculating the sample size for prevalence studies. Archives of Orofacial Sciences. 2006;1:9-14.

[23] Grönberg H, Adolfsson J, Aly M, . Prostate cancer screening in men aged 50-69 years (STHLM3): a prospective population-based diagnostic study. Lancet Oncol. 2015;16(16):1667-1676. doi:10.1016/S1470-2045(15)00361-7 26563502

[24] Eklund M, Nordström T, Aly M, . The Stockholm-3 (STHLM3) model can improve prostate cancer diagnostics in men aged 50–69 years compared with current prostate cancer testing. *Eur Urol Focus**.* 2018;4(5):707-710. doi:10.1016/j.euf.2016.10.009 28753803

[25] Thompson IM, Pauler Ankerst D, Chi C, . Operating characteristics of prostate-specific antigen in men with an initial PSA level of 3.0 ng/ml or lower. *JAMA**.* 2005;294(1):66-70. doi:10.1001/jama.294.1.66 15998892

[26] Thompson IM, Pauler DK, Goodman PJ, . Prevalence of prostate cancer among men with a prostate-specific antigen level < or =4.0 ng per milliliter. N Engl J Med. 2004;350(22):2239-2246. doi:10.1056/NEJMoa031918 15163773

[27] Dobrow MJ, Hagens V, Chafe R, Sullivan T, Rabeneck L. Consolidated principles for screening based on a systematic review and consensus process. CMAJ. 2018;190(14):E422-E429. doi:10.1503/cmaj.171154 29632037 PMC5893317

[28] Ran T, Cheng C-Y, Misselwitz B, Brenner H, Ubels J, Schlander M. Cost-effectiveness of colorectal cancer screening strategies—a systematic review. Clin Gastroenterol Hepatol. 2019;17(10):1969-1981.e15. doi:10.1016/j.cgh.2019.01.014 30659991

[29] Brown J, Bryan S, Warren R. Mammography screening: an incremental cost effectiveness analysis of double versus single reading of mammograms. BMJ. 1996;312(7034):809-812. doi:10.1136/bmj.312.7034.809 8608287 PMC2350705

