# Supplementary material for: Population-Based Prostate Cancer Screening With Magnetic Resonance Imaging or Ultrasonography: The IP1-PROSTAGRAM Study
Source: JAMA Oncol. 2021 Feb 11;7(3):395–402. doi: 10.1001/jamaoncol.2020.7456 (PMC7879388; doi:10.1001/jamaoncol.2020.7456)
Supplement: Supplement. — eMethods. Supplemental Methods eTable 1. PSA Levels <3ng/ml in Men With an MRI Score 3-5 & Significant Cancer on Biopsy eTable 2. Agreement in MRI Scores Between Primary Readers and a Secondary Reader eTable 3. Agreement in US Scores Between Primary Reader and a Secondary Reader eTable 4. Adverse Events eFigure 1. A Screen-Negative MRI eFigure 2. A Screen-Positive MRI and Screen-Negative Ultrasound eFigure 3. Venn Diagrams Demonstrating Overlap of Clinically Significant Cancers Identified by Each Screening Test eFigure 4. Flowchart of MRI Score 3-5 and Detection of Clinically Significant Cancer eFigure 5. Flowchart of MRI Score 4-5 and Detection of Clinically Significant Cancer eFigure 6. Flowchart of US Score 3-5 and Detection of Clinically Significant Cancer eFigure 7. Flowchart of US Score 4-5 and Detection of Clinically Significant Cancer eFigure 8. Flowchart of PSA ≥ 3ng and Detection of Clinically Significant Cancer [file jamaoncol-e207456-s001.pdf]

Supplementary Online Content

Eldred-Evans D, Burak P, Connor MJ, et al. Population-based prostate cancer screening with magnetic resonance imaging or ultrasonography: the IP1-PROSTAGRAM study. *JAMA Oncol.* Published online February 11, 2021. doi:10.1001/jamaoncol.2020.7456

eMethods. Supplemental Methods

eTable 1. PSA Levels <3ng/ml in Men With an MRI Score 3-5 & Significant Cancer on Biopsy

eTable 2. Agreement in MRI Scores Between Primary Readers and a Secondary Reader

eTable 3. Agreement in US Scores Between Primary Reader and a Secondary Reader

eTable 4. Adverse Events

eFigure 1. A Screen-Negative MRI

eFigure 2. A Screen-Positive MRI and Screen-Negative Ultrasound

eFigure 3: Venn Diagrams Demonstrating Overlap of Clinically Significant Cancers Identified by Each Screening Test

eFigure 4. Flowchart of MRI Score 3-5 and Detection of Clinically Significant Cancer

eFigure 5. Flowchart of MRI Score 4-5 and Detection of Clinically Significant Cancer

eFigure 6. Flowchart of US Score 3-5 and Detection of Clinically Significant Cancer

eFigure 7. Flowchart of US Score 4-5 and Detection of Clinically Significant Cancer

eFigure 8. Flowchart of PSA ≥ 3ng and Detection of Clinically Significant Cancer

This supplementary material has been provided by the authors to give readers additional information about their work.

eMethods: Supplemental Methods

Primary care searches

Primary care practices ran database searches using pre-defined eligibility criteria. The filters for the searches excluded men with:

- A prostate-specific antigen level or prostate MRI in the last 2 years
- An infection of the urinary tract or prostatic inflammatory disease in the last 6 months
- A previous diagnosis of tumour of prostate or treatment for prostate cancer
- Contraindications to PSA or MRI such as a needle phobia, claustrophobia, MRI incompatible devices, BMI 40kg/m^2, glaucoma, low mobility, degenerative neurological disease or patients on home oxygen
- Contraindications to prostate biopsy such as congenital bleeding disorders or anticoagulation
- Co-morbidities which reduce life expectancy to <10 years such as metastatic cancer, person's on palliative care register, Acquired immunodeficiency syndrome, Congestive Heart failure, Chronic obstructive pulmonary disease (Medical Research Council (MRC) dyspnoea scale 4-5), myocardial infarction or unstable angina in last 12 months, Portal Hypertension/Liver cirrhosis, Chronic kidney disease 4 or 5

A general practitioner further screened lists to remove any men with other co-morbidities and/or frailty which would have meant that an individual's life expectancy would limit their benefit from screening or other reasons why it may be inappropriate for the patient to receive an invitation. The process for letter invitation to take part involved uploading the final patient list to an online mailing company (Docmail). All men in the list were sent an invitation letter with an information leaflet or an SMS depending on the GP practice policy. An expression of interest in the study was via a study telephone line, email or website address.

Local Community Recruitment

Previous screening trials have had a low screening uptake among certain racial and ethnic groups. IP1-PROSTAGRAM aimed to achieve participant recruitment which is more representative across racial ethnic risk groups for prostate cancer. Therefore, attempts were made to maximise racial and ethnic minority recruitment to ensure it was more representative of the local community around the West London site which has a high racial and ethnic diversity.

The key elements of this recruitment strategy were developed with input from members of the local community prior to commencement of the trial. It involved a multi-modal recruitment strategy for informing eligible men about the trial using:

1. Posters and flyers in areas frequented by our target group (e.g. community groups, libraries, gyms) and in local newspapers
2. Community group leaders who shared the study website via social media
3. Media coverage on local radio stations

Persons who expressed an interest had the option of contacting the recruitment centre by telephone or via the study website for a web-app-facilitated survey to screen for eligibility. The recruitment centre was managed by clinical study officers from the National Institute for Health Research (NIHR) who completed the eligibility check against the inclusion and exclusion criteria described below.

© 2021 Eldred-Evans et al. *JAMA Network Open.*

Inclusion Criteria

1. Men aged between 50 and 69 years inclusive at the time of study entry<br>2. Participants must be fit to undergo all procedures listed in the protocol<br>3. Estimated life expectancy of 10 years or more<br>4. An understanding of the English language sufficient to understand written and verbal information about the trial and consent process<br>5. Participants must be willing and able to provide written informed consent

Exclusion Criteria

1. Previous PSA test or prostate MRI within the prior two years of screening/consent visit<br>2. Evidence of a urinary tract infection or history of acute prostatitis within the last 6 months<br>3. Previous history of prostate cancer, prostate biopsy or treatment for prostate cancer (interventions for benign prostatic hyperplasia/bladder outflow obstruction is acceptable)<br>4. Any potential contraindication to MRI, including but not limited to:<br>a. Devices or metallic foreign bodies such as pacemakers, implantable defibrillators, neurostimulators, cochlear implants, coronary stents, prosthetic heart valves, aneurysm clips and other intravascular devices<br>b. Previous history of hip replacement surgery, metallic hip replacement or extensive pelvic orthopaedic metal<br>c. Claustrophobia<br>5. Any potential contraindication to prostate biopsy<br>6. Dementia or altered mental status that would prohibit the understanding or rendering of informed consent<br>7. Any other medical condition precluding procedures described in the protocol

© 2021 Eldred-Evans et al. *JAMA Network Open.*

MRI Protocol

Sequence	Plane	TR (ms)	TE (ms)	Aver-ages	FA (degree)	WFS (pix)	BW (Hz/Px)	FoV (mm)	Phase FOV (% of FOV)	Over-sampling (% of FOV)	Phase enc. direction	Slice thickness (mm)	Slice gap (% of slice thickness)	TSE/EPI factor	FS method	Matrix	Phase res. (% of matrix)	Recon. voxel size (mm)	Sequence duration (mm:ss)
**3T SIEMENS MAGNETOM Verio syngo MR B17**																			
**Localiser**	Multiplanar	1000	92	1	150	1.2	349	400	100	20	Multiple	7	100	256		256	100	1.6x1.6x7	00:15
**T2 TSE**	Sagittal	7000	101	3	Min 150	2.0	200	200	100	43	H>F	3	20	25		320	80	0.8x0.6x3	02:57
**T2 TSE**	Axial	7000	108	2	Min 150	1.1	200	363	100	100	R>L	3	0	24		320	80	0.8x0.6x3	02:43
**DWI** (b0, 150, 400, 1000)	Axial	8500	80	3		0.2	1698	250	100	30	A>P	3	0	128	SPAIR	128	100	2 x 2 x 3	04:42
**DWI (b1500)**	Axial	9100	85	7		0.2	1698	250	100	30	A>P	3	0	128	SPAIR	128	100	2 x 2 x 3	03:40
**1.5T SIEMENS MAGNETOM Aera**																			
**Localiser**	Multiplanar	1000	93	1	180	0.4	501	400	100	20	Multiple	7	100	256		256	100	1.6x1.6x7	00:11
**T2 TSE**	Sagittal	5280	125	3	Min 150	1	200	200	100	100	H>F	3	20	23		320	80	0.6x0.6x3	03:17
**T2 TSE**	Axial	4590	135	3	Min 150	1	200	200	100	100	R>L	3	0	23		320	80	0.6x0.6x3	02:51
**DWI (b0, 150, 400, 1000)**	Axial	7500	67	2, 3, 4, 5		0.1	1507	250	100	30	A>P	3	0	128	SPAIR	128	100	2 x 2 x 3	05:23
**DWI (b1500)**	Axial	7500	68	9		0.1	1502	250	100	30	A>P	3	0	128	SPAIR	128	100	2 x 2 x 3	04:00
All scans were performed with intravenous administration of 20 mg hyoscine butylbromide. If contraindicated, 1 mg of glucagon hydrochloride was used intravenously. If both bowel relaxants were contra-indicated, no medication was used																			

Quality control

Scans with poor quality images were repeated and if the quality of the diffusion-weighted imaging sequences was compromised by air, participants were offered a rectal flatus tube to decompress the rectum. To reduce motion artefact from bowel peristalsis, an antispasmodic agent was administered to all participants.

© 2021 Eldred-Evans et al. *JAMA Network Open.*

MRI Reporter Experience

	Radiologist 1	Radiologist 2	Radiologist 3
Number of years' experience	9	8	> 15 years
Number of prostate MRIs reported per annum	1000	300	300

Ultrasonographer Experience

	Ultrasonographer 1	Ultrasonographer 2
Number of years' experience	4	2
Number of prostate US performed per annum	300	120

Ultrasound Scoring

B-mode Score (from Xie et al, 2018[19])
▪ Score 1: Benign (homogeneous hyperechogenicity of the peripheral zone (PZ) and intermediate echogenicity of the transition zone (TZ)).<br><br>▪ Score 2: Probably benign (minimal heterogeneity, linear or wedge-shaped hypoechogenicity in the PZ; circumscribed hypoechoic or heterogeneous encapsulated nodule[s] in the TZ).<br><br>▪ Score 3: Indeterminate (contour asymmetry; ill-defined echotexture abnormality or non-circumscribed, rounded, moderate hypoechogenicity in the PZ; heterogeneous echogenicity with obscured margins in the TZ; includes others that do not qualify as 2, 4, or 5).<br><br>▪ Probably malignant (focal contour bulge; focal ill-defined borders between the PZ and TZ; circumscribed, homogenous moderate hypoechoic mass confined to the prostate [<1.5 cm in greatest dimension] with/without microcalcifications in PZ; lenticular or non-circumscribed, homogeneous, moderately hypoechoic [<1.5 cm in greatest dimension] in TZ).<br><br>▪ Score 5: Malignant (obvious focal contour bulge or definite extraprostatic extension/ invasive behavior; diffusely ill-defined borders between the PZ and TZ; focal hypoechoic mass[es] [≥1.5 cm in greatest dimension] with/without microcalcifications in the PZ; diffuse hypoechoic in PZ with/without ill-defined borders between the PZ and TZ; lenticular or non-circumscribed, homogeneous, moderately hypoechogenic [≥1.5 cm in greatest dimension] in the TZ).

© 2021 Eldred-Evans et al. *JAMA Network Open.*

Shearwave Elastography Score (adapted from WFUMB guidelines[20])
▪ Score 1: Normal appearance (homogeneous elasticity pattern, the peripheral and central zones are evenly shaded in blue)<br>▪ Score 2: Probably normal (symmetric heterogeneous elasticity pattern not corresponding to a hypoechoic area)<br>▪ Score 3: Indeterminate (focal asymmetric area of high stiffness not related to a hypo-echoic lesion)<br>▪ Score 4: Probably carcinoma (a high mean elasticity value within the centre of a hypo-echoic lesion)<br>▪ Score 5: Definitely carcinoma (a high mean elasticity value within an entire hypo-echoic lesion)

Sample Size estimation

The sample size estimate for the primary outcome was calculated using the formula described by Naing et al. with the following assumptions to determine an estimate of the prevalence of screen-positive MRI:

(1) In the general population, 89.6% of men have a PSA <3ng/ml and 10.4% have a PSA ≥ 3ng/ml

(2) In a population of men with a PSA <3ng/ml, a prevalence of clinically-significant prostate cancer of 2.2%

(3) MRI sensitivity and specificity were estimated as 74-93% and 41-88%[6,20], respectively

(4) In men with a PSA <3ng/ml, a prevalence of screen-positive MRI of 13.4-59.8%[6,20]

(5) In a population of men with PSA ≥ 3ng/ml, a prevalence of screen-positive MRI of 73%[6]

Based on these assumptions, a minimum target of 270 participants (low prevalence [19.6%] of screen-positive MRI) and a maximum target of 406 participants (high prevalence [61.1%] of screen-positive MRI) were calculated to provide a ±5% precision estimate at a two-sided significance level of 0.05, allowing for a 10% dropout rate.

© 2021 Eldred-Evans et al. *JAMA Network Open.*

eTable 1: PSA levels <3ng/ml in men with an MRI score 3-5 & significant cancer on biopsy

	PSA Level (ng/ml)
Patient 1	0.69
Patient 2	0.90
Patient 3	1.02
Patient 4	1.15
Patient 5	1.70
Patient 6	1.77
Patient 7	2.13
Patient 8	2.86

eTable 2: Agreement in MRI scores between primary readers and a secondary reader

		Secondary reader				
		**1 - 2**	**3**	**4**	**5**	**Total**
**Primary readers**	**1 - 2**	21	2	2	1	26
	**3**	14	9	2	1	26
	**4**	9	5	5	0	19
	**5**	2	1	1	3	7
	**Total**	46	17	10	5	78

Red indicates agreement between PIRADS scores

Green shading indicates concordant scores, where management decision to perform biopsy would not have changed.

Blue shading indicates discordant scores, where management decision to perform biopsy would have changed.

* Men with MRI score which was upgraded by the third independent reader were reviewed clinically provided they had not already undergone a biopsy as part of the trial. There were three additional participants with a screen-positive MRI (score 4-5) according to the secondary report and these participants underwent biopsy which were all benign.

Interobserver Agreement using MRI Thresholds ≥ 3 or ≥ 4 (N = 78)

MRI score	Agreement (%)	Expected agreement (%)	Kappa statistic	Standard error	95% confidence interval	t	Pr > |t|
**3-5**	61.5%	47.0%	0.274	0.093	0.089 to 0.460	2.94	0.004
**4-5**	70.5%	60.3%	0.258	0.113	0.033 to 0.484	2.28	0.03

© 2021 Eldred-Evans et al. *JAMA Network Open.*

eTable 3: Agreement in US scores between primary reader and a secondary reader

		Secondary reader				
		1 - 2	3	4	5	Total
Primary reader	1 - 2	19	0	7	0	26
	3	7	4	13	2	26
	4	4	1	17	1	23
	5	0	0	3	0	3
	Total	30	5	40	3	78

Red indicates agreement between US scores

Green shading indicates concordant scores, where management decision to perform biopsy would not have changed.

Blue shading indicates discordant scores, where management decision to perform biopsy would have changed.

Interobserver Agreement using US Thresholds ≥ 3 or ≥ 4 (N = 78)

US score	Agreement (%)	Expected agreement (%)	Kappa statistic	Standard error	95% confidence interval	t	Pr > |t|
3-5	76.9%	53.9%	0.500	0.102	0.297 to 0.703	4.90	<0.001
4-5	65.4%	48.3%	0.331	0.096	0.140 to 0.522	3.45	0.001

eTable 4: Adverse Events

Adverse Events by procedure	N = 26
**MRI**	
Procedure related anxiety/pain	2
Sensation of over-heating	1
**Ultrasound**	
Procedure related anxiety/pain	4
Mild allergic reaction to latex	1
**PSA**	
Superficial Infection	1
**Other study procedures (e.g. prostate biopsy)**	
Haematuria	5
Haematospermia	4
Procedure related pain	1
Other	4
Urinary Tract Infection	2
Lower urinary tract symptoms	2
**Unrelated to study procedures**	
Cold-like symptoms	1

© 2021 Eldred-Evans et al. *JAMA Network Open.*

eFigure 1: A screen-negative MRI

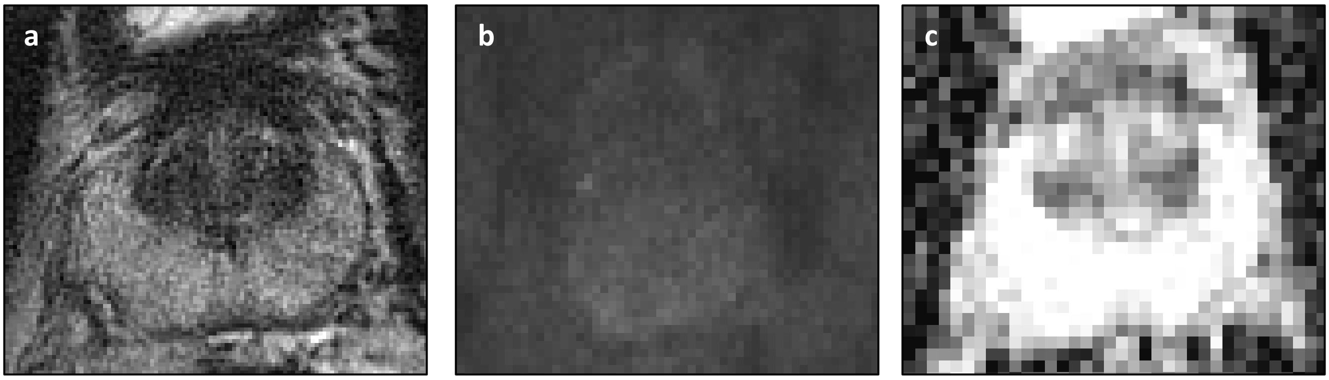

55-year-old man with a screen-positive PSA 8.60ng/ml (a) Axial T2-weighted image (b) Diffusion-weighted imaging (b value = 1500 s/mm) (c) Apparent diffusion coefficient map through the mid-gland showing no abnormalities. The prostate volume was 34ml. The 12-core systematic biopsy did not identify any prostate cancer.

eFigure 2: A screen-positive MRI and screen-negative ultrasound

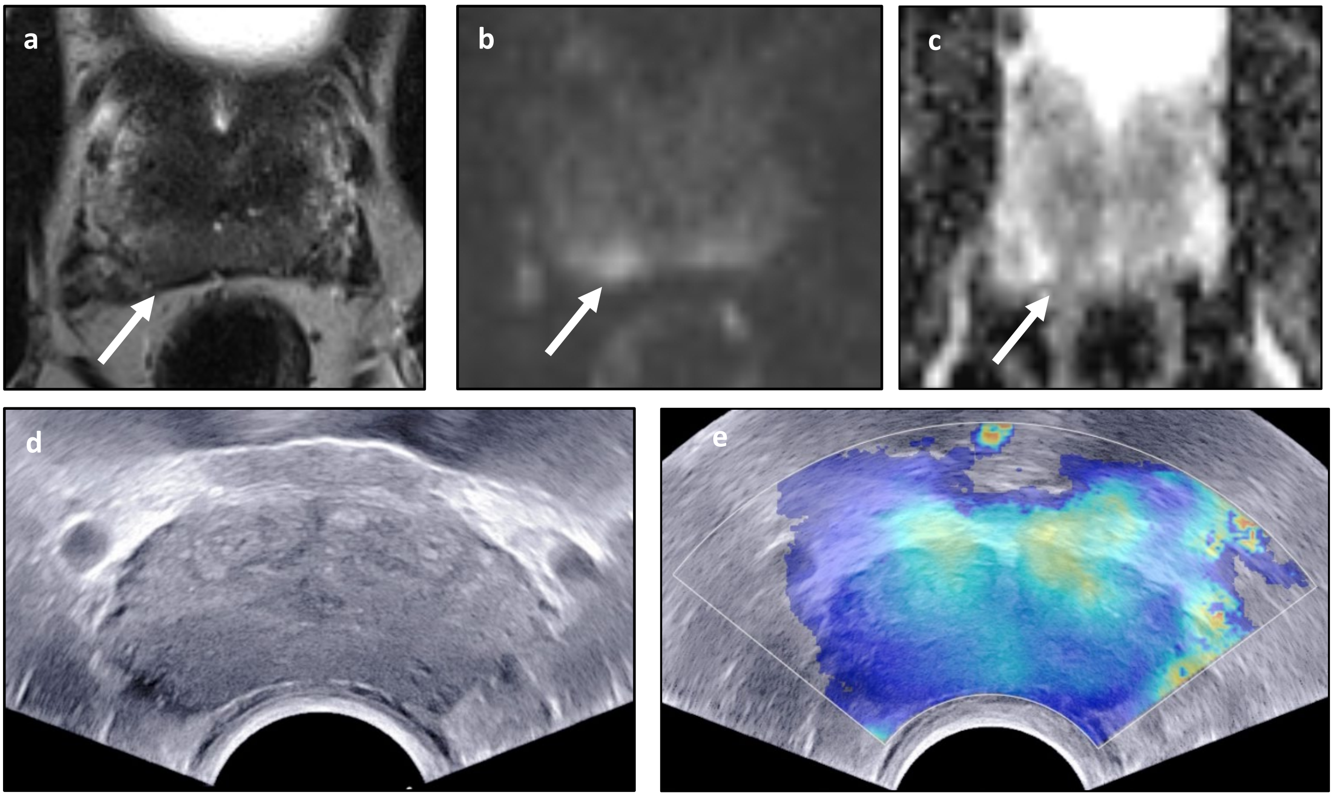

58-year-old man with a screen-negative PSA 1.02ng/ml, a screen-negative ultrasound and a screen-positive MRI which was scored by both primary and secondary reporters as 4 out of 5 at the right base (arrows). The MRI sequences (a,b,c) are identical to eFigure 1 with a high b-value 1500s/mm. (d) Transverse b-mode ultrasound and (e) shear wave elastography at the base of the prostate showing a homogenous peripheral zone with mean elastography values 22.4kpa. This was reported as 1 out of 5 (screen-negative ultrasound). The MRI-ultrasound image-fusion targeted biopsy found clinically significant prostate cancer (Gleason 3+4 with a maximum cancer length 7mm) in all MRI targeted biopsy cores.

© 2021 Eldred-Evans et al. *JAMA Network Open.*

eFigure 3: Venn Diagrams Demonstrating Overlap of Clinically Significant Cancers Identified by Each Screening Test

A: Score of 3 to 5

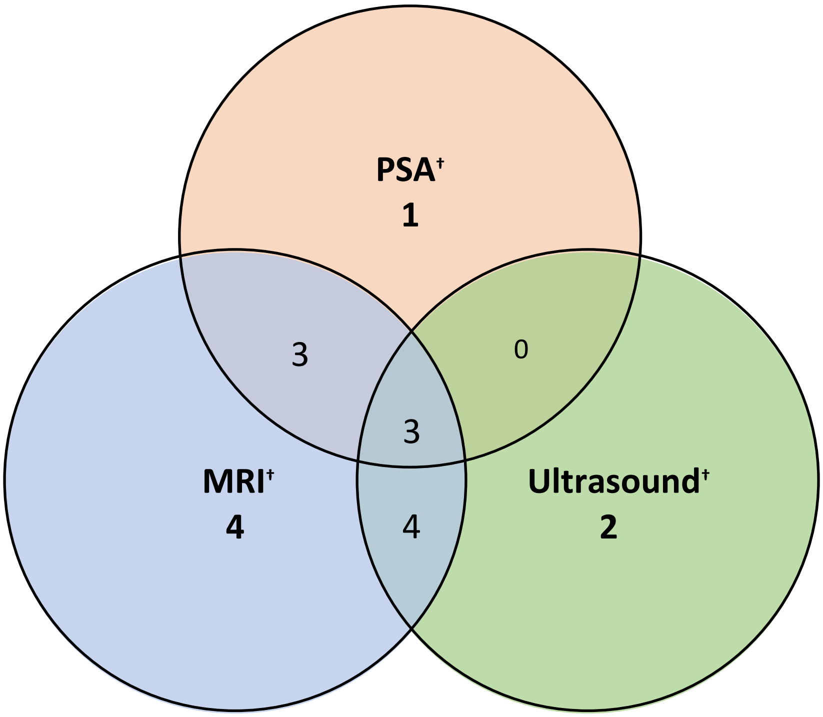

*Clinically significant cancer defined as Gleason ≥3+4 (ISUP ≥2)
† Screen-positive test defined as PSA ≥ 3ng/ml, MRI-Score 3-5, Ultrasound Score 3-5

B: Score of 4 to 5

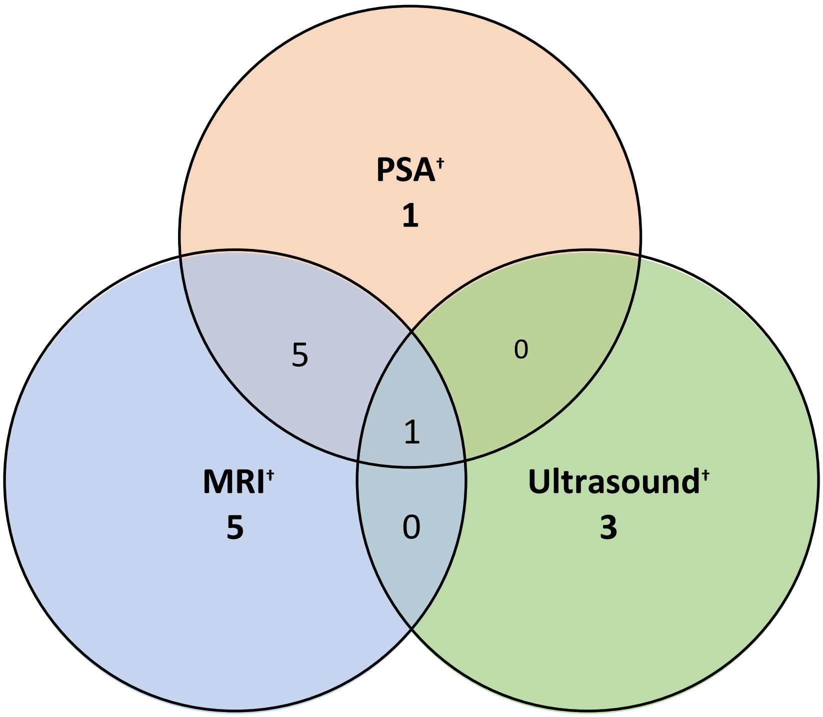

† Screen-positive test defined as PSA ≥ 3ng/ml, MRI-Score 4-5, Ultrasound Score 4-5

© 2021 Eldred-Evans et al. *JAMA Network Open.*

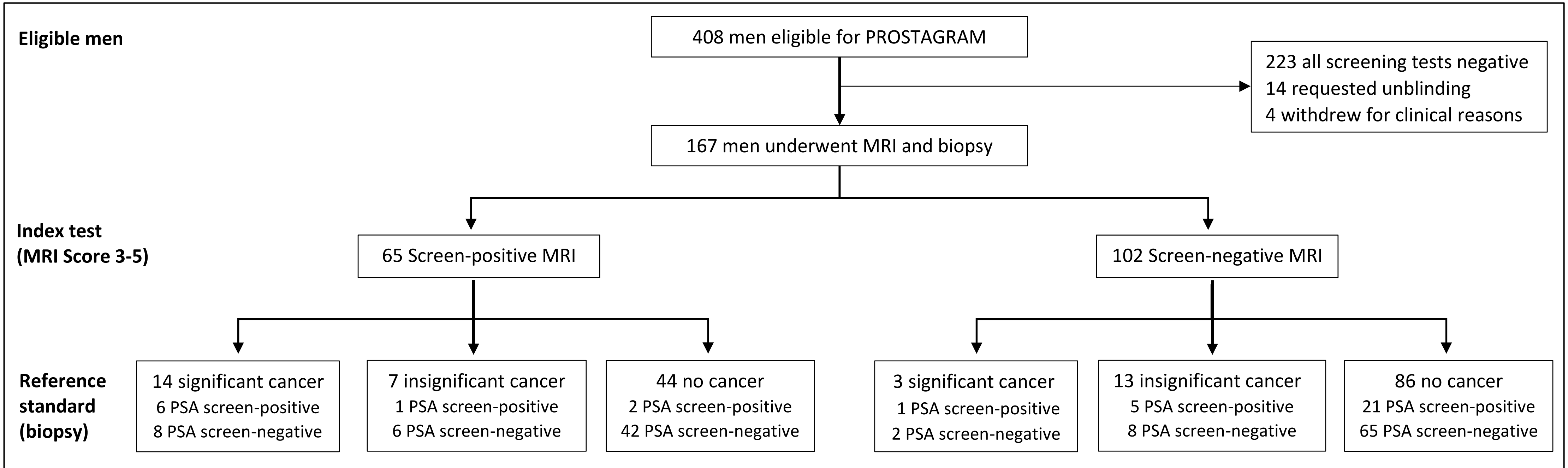

eFigure 4: Flowchart of MRI score 3-5 and detection of clinically significant cancer

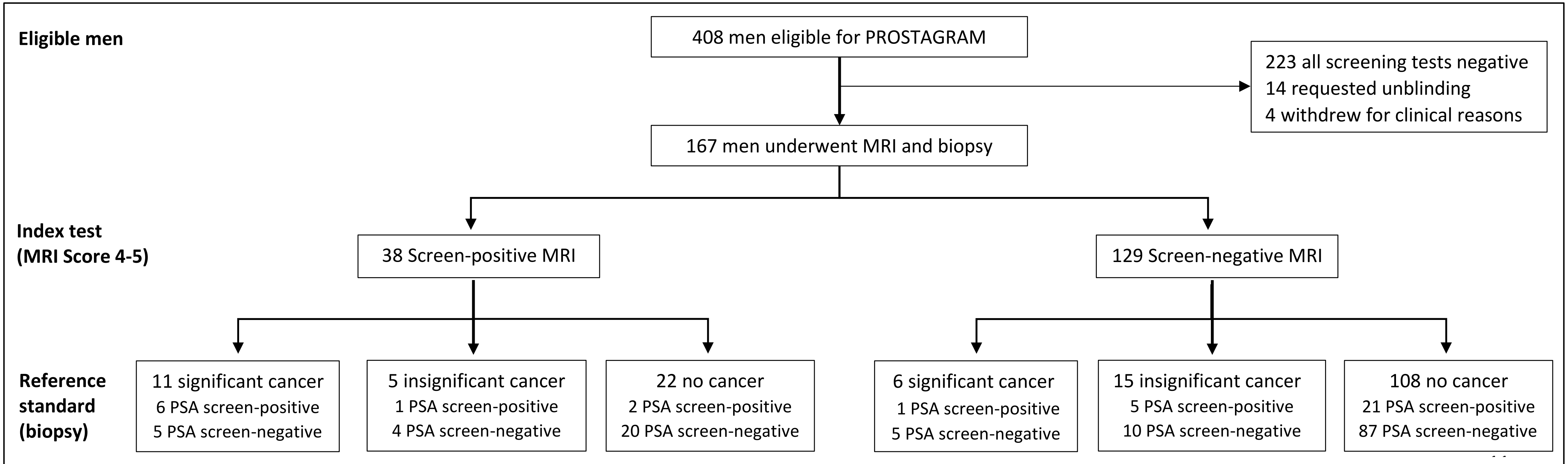

eFigure 5: Flowchart of MRI score 4-5 and detection of clinically significant cancer

© 2021 Eldred-Evans et al. *JAMA Network Open.*

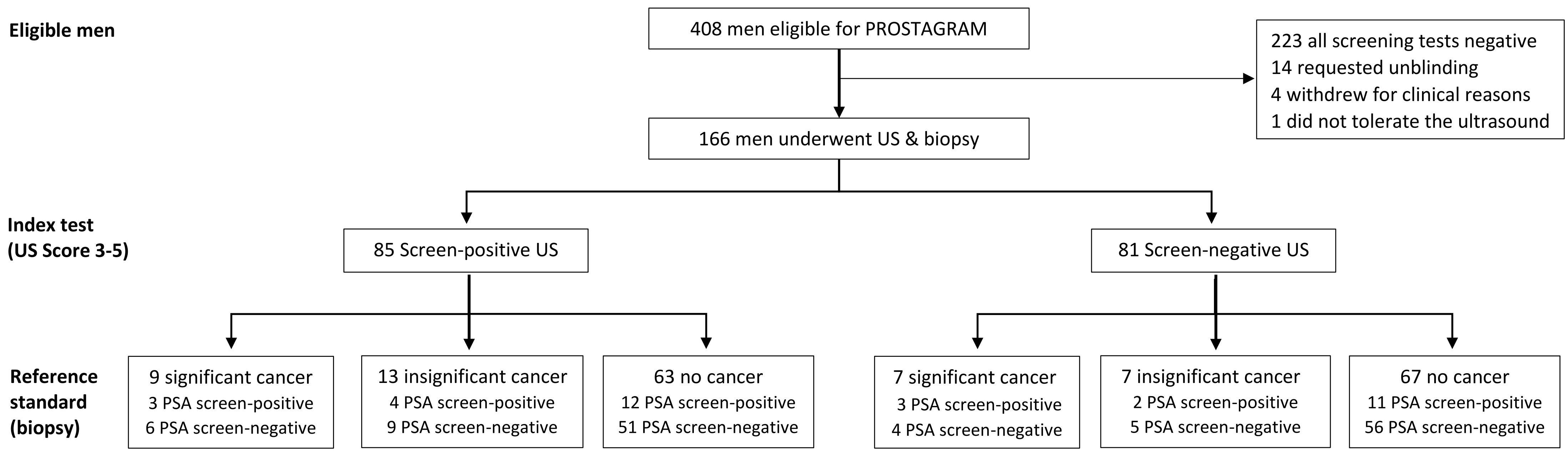

eFigure 6: Flowchart of US score 3-5 and detection of clinically significant cancer

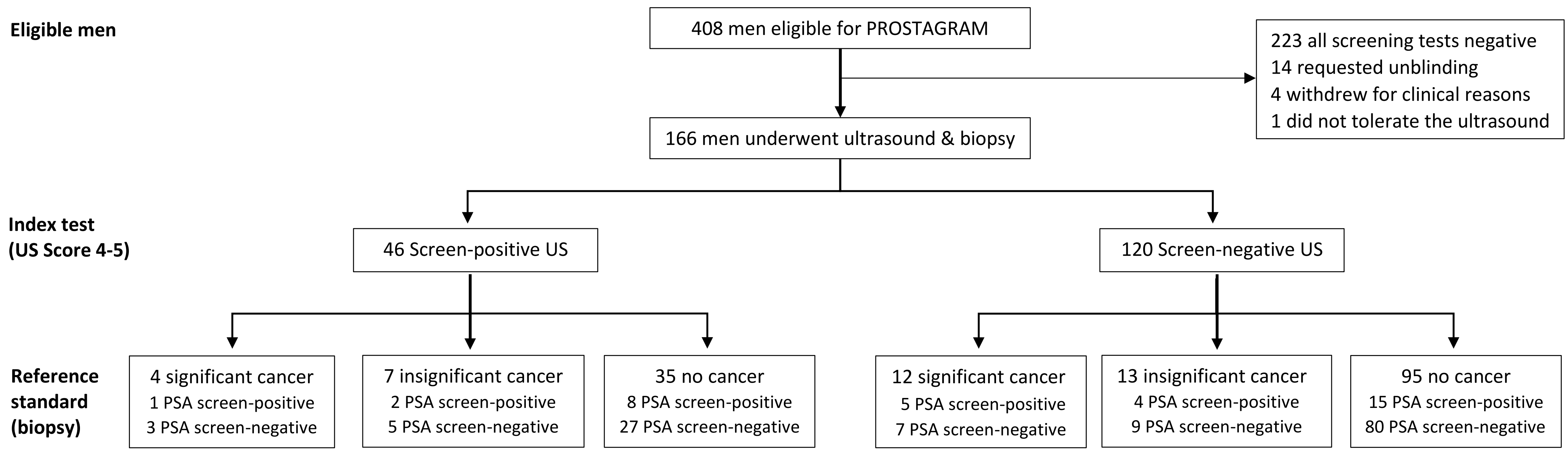

eFigure 7: Flowchart of US score 4-5 and detection of clinically significant cancer

© 2021 Eldred-Evans et al. *JAMA Network Open.*

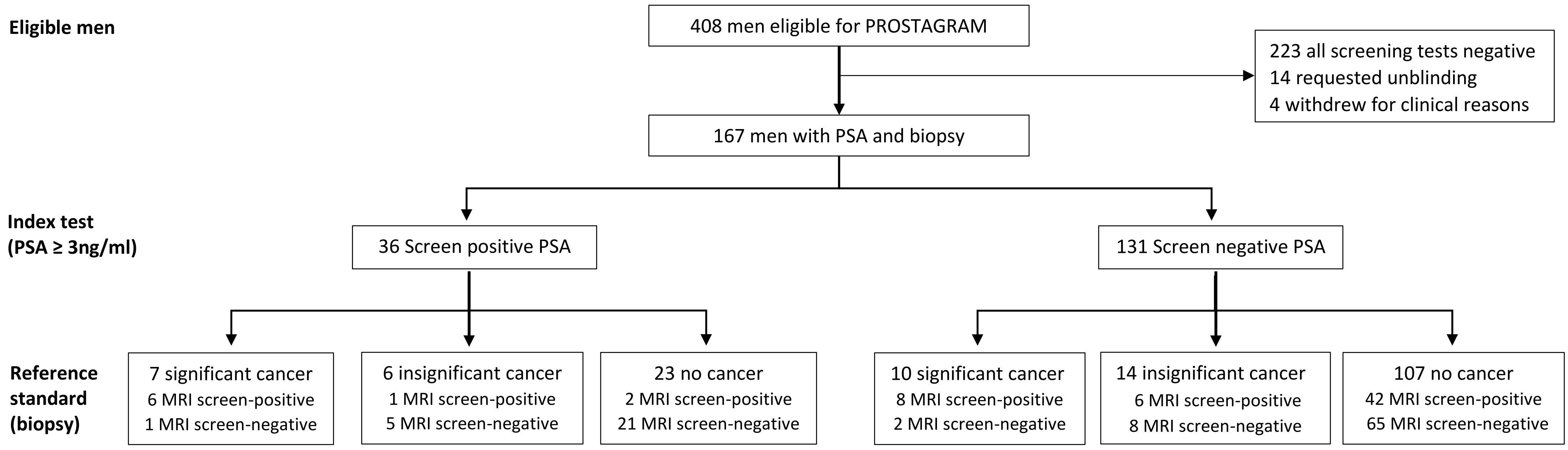

eFigure 8: Flowchart of PSA ≥ 3ng and detection of clinically significant cancer

© 2021 Eldred-Evans et al. *JAMA Network Open.*
